# Complete Genome Sequence of Raoultella electrica 1GB (DSM 102253^T^), Isolated from Anodic Biofilms of a Glucose-Fed Microbial Fuel Cell

**DOI:** 10.1128/MRA.00800-19

**Published:** 2019-10-24

**Authors:** Simone Thiel, Boyke Bunk, Cathrin Spröer, Jörg Overmann, Dieter Jahn, Rebekka Biedendieck

**Affiliations:** a Institute of Microbiology and Braunschweig Integrated Center of Systems Biology, Technische Universität Braunschweig, Braunschweig, Germany; b Leibniz Institute DSMZ-German Collection of Microorganisms and Cell Cultures, Braunschweig, Germany; University of Delaware

## Abstract

The type strain Raoultella electrica 1GB (DSM 102253^T^) was isolated from anodic biofilms of glucose-fed microbial fuel cells. The fully assembled, closed, circular 5.27-Mb genome and corresponding 0.52-Mb plasmid DNA sequences were elucidated. Potential electron transfer and pathogenicity mechanisms were deduced.

## ANNOUNCEMENT

The genome sequence of the Gram-negative bacterium Raoultella electrica 1GB, which was isolated from an anodic biofilm of a glucose-fed microbial fuel cell (1), was determined. So far, only genome sequences for the related species Raoultella ornithinolytica, Raoultella planticola, and Raoultella terrigena have been published (2–5). *R. electrica* 1GB (DSM 102253^T^) was provided by the Leibniz Institute DSMZ and cultured in lysogenic broth (LB). DNA was isolated using the DNA binding column Genomic-tip 100/G (Qiagen, Hilden Germany). A SMRTbell template library was prepared according to the instructions from Pacific Biosciences (Menlo Park, CA, USA). Genomic DNA was sheared using g-TUBEs from Covaris (Woburn, MA, USA), according to the manufacturer’s instructions. One single-molecule real-time (SMRT) cell was sequenced on the PacBio RS II system (Pacific Biosciences) by taking one 240-minute movie, leading to 108,718 reads. Paired-end libraries for sequencing on an Illumina platform were prepared by applying the Nextera XT DNA library preparation kit (Illumina, San Diego, CA, USA), with modifications (6), leading to 3.6 million reads of 2 × 150 bp. Samples were sequenced on a NextSeq 500 platform. Long-read genome assembly was performed by applying the RS_HGAP_Assembly.3 protocol included in SMRT Portal version 2.3.0, using default parameters. All replicons were circularized; in particular, artificial redundancies at the ends of the contigs were removed, and adjustment to *dnaA* (chromosome) or *repA* or *parB* (plasmids) was performed. Identification of redundancies and of replication genes has been done based on BLAST, and circularization and rotation to the replication genes has been performed by genomecirculator.jar tool (https://github.com/boykebunk/genomefinish). Error correction was performed by mapping Illumina short reads onto the finished genome using Burrows-Wheeler Alignment (bwa) 0.6.2 in paired-end (sampe) mode, with default settings (7), and subsequent variant and consensus calling was performed using VarScan 2.3.6 (8). A consensus concordance of QV60 was confirmed. Automated genome annotation was carried out using Prokka 1.8 (9). The genome of *R. electrica* consists of a circular chromosome of 5,266,426 bp and five circular plasmids of 303,830 bp, 92,370 bp, 83,988 bp, 35,253 bp, and 3,336 bp in size. The G+C contents were determined to be 55% (chromosome), 48% (plasmid 1), 52% (plasmid 2), 51% (plasmid 3), 42% (plasmid 4), and 46% (plasmid 5). According to genome coverage analysis, plasmids 1 to 5 have copy numbers of 1, 2, 1, 3, and 3, respectively, at an overall chromosomal coverage of 130×. There are 5,654 predicted genes within the complete genome, including 5,390 genes for proteins, with 503 carrying signal peptides, 25 for rRNAs, 87 for tRNAs, 133 for noncoding RNAs, and, finally, 2 CRISPR structures. *c*-Type cytochromes are usually the key players in direct extracellular electron transfer (10). Surprisingly, no *c*-type cytochromes were found in the genome of *R. electrica*. Correspondingly, no genes coding for proteins that are known for the transfer of electrons through the periplasm and outer membrane, such as *mtrC*, *omcA*, *mtrA*, *mtrB,* and *cymA,* were found (11). Other electron transport components, including Rnf complex proteins, are present. *R. ornithinolytica*, *R. planticola,* and *R. terrigena* cause urinary tract and gastrointestinal infections (12–14). *R. electrica* was found to be resistant to several antibiotics (15). The genome contains genes for chloramphenicol acetyltransferase, spectinomycin tetracycline efflux pump, and different penicillin-binding proteins. Genes for hemolysin transport (*shlB,* electrica_02770 and electrica_04075), expression modulation (*hha*, electrica_03875 and electrica_03970), and maturation (*hlyC*, electrica_02771) (16) were found. Filamentous hemagglutinin (FhaB, electrica_02772, electrica_02779, electrica_02786, and electrica_04076), an *S*-fimbrial adhesion protein (Sfa, electrica_00816 to electrica_00818), type IV secretion system component, and multiple mercuric and tellurite resistance (17) proteins are encoded by the genome and the various plasmids.

### Data availability.

The genome sequence has been deposited at NCBI GenBank under accession numbers CP041247 to CP041252. The versions described in this paper are the first versions, CP041247.1 to CP041252.1. Raw sequence reads have been submitted to the NCBI SRA under the accession numbers SRR9665439 (PacBio) and SRR9665440 (Illumina).
